# Letter from Accoucheur.—No. 5

**Published:** 1846-04-01

**Authors:** 


### Letter from Accoucheur.—No. 5.

For the Buffalo Medical Journal.

No incident in the parturient state gives the Accoucheur more anxiety and apprehension than uterine hemorrhage before and after delivery; and it will be most happy for him if he have often contemplated its probable occurrence, and have decided beforehand, how to act with promptitude and decision, prospectively as it were, in the emergency.

Its access is usually sudden and without premonition; often profuse and perilous. Before delivery it may arise from premature detachment of a part, or the whole of the placenta; or from its attachment to, or near the cervix uteri. In either case, when the hemorrhage is profuse the safety of the mother demands immediate delivery. If it appear on examination that the condition of the organs will not justify an attempt to effect delivery, the accoucheur may expedite it by provoking the uterus to sufficient expulsive action by irritating the tincae as has been described before; or by plugging the vagina he may suppress the hemorrhage until pains return, or the condition of the organs will enable him to accomplish safely the delivery. As long as the uterus can be provoked to act, the patient will not die of hemorrhage; but when the pains cease, and the uterus can no longer be made to contract, there should be no delay—no reliance should be placed on the use of refrigerants, or astringents, or styptics, or on ergot, supposed by some—and to a certain extent the opinion still obtains—to exert a specific influence on the uterus in parturition. After repeated experiments made years ago with ergot gathered fresh from the fields by myself, and after having witnessed its exhibition by the sanguine advocates for its use, I aver that I have never seen, in a single instance, any decisive, unequivocal effect from its administration; and I am constrained to believe it to be inert, and useless, having no more power on the uterus, under any circumstances, than the same amount of saw-dust. In the present state of opinion, inert and useless as I believe it to be, I would neither deprecate nor condemn its use, for in lingering and protracted labor I have often happily succeeded in soothing the impatience and raising the despondency of the patient by *cautiously* administering what was *suspected* to be ergot. In a word, ergot, the "Pulvis Parturiens"!! I consider a harmless humbug; destined perhaps to give way and yield the palm to Mesmerism—another humbug of "purer ray" or "deeper dye."

When the pains have ceased from hemorrhage, and langor, exhaustion, and approaching syncope admonish the accoucheur of the necessity of immediate delivery, if he cannot avail himself of the vectis, the forceps, blunt-hook, or crotchet, he will find little or no resistance or difficulty in proceeding to turn the child, and deliver *by a foot*; and if the uterus do not then immediately contract, he will plug the vagina and compress the abdomen.

I was called to Mrs. W., the mother of a numerous family, and found her on the bed, pale and apparently lifeless, and deluged in blood; her friends standing around aghast. The question arose instinctively to my lips, is she dead? Proceeding instantly to an examination, to my consternation and horror, I found the arm of a foetus protruded to the shoulder ; thrusting my fingers into the axilla of the child, I raised the shoulders without resistance, and in doing it a foot slid into my hand, which I grasped, brought it down and accomplished the delivery,—foetus and placenta simultaneously, in less time than I have taken to record it. Mrs. W. recovered.

I was called to the wife of a Welch Weaver, and found her on a straw pallet, pale, cold, pulseless, and insensible, surrounded by a bevy of her country-women, of whom I could learn nothing. Proceeding to an examination, I encountered the placenta, expelled, and the cord was broken ; tracing the cord to the head of the child resting on the brim of the pelvis, I put it aside and carried my hand forward in search of a foot, which I found without difficulty, brought it down and accomplished the delivery. This poor woman recovered, but it was many days before she could be made conscious that she had become a mother.

Extreme cases to be sure are not of every day occurrence, nevertheless they occur often enough to render every accoucher of middle age, somewhat familiar with them.—Suppose, and it is "a question to be asked"—suppose in the two cases above described, (more might be adduced,) instead of resorting to the *ultimâ ratio*, delivery — the vagina had been plugged (if practicable) with ice, or the abdomen covered with a cold wet napkin, or acetate of lead and opium had been administered, ergot in substance, or decoction.—What probably would have been the result? Does it need "any ghost from the grave" to answer the question? In all cases of uterine hemorrhage before delivery when the accoucheur shall have decided on immediate delivery, if he cannot avail himself of instruments, he will proceed to introduce his hand without regarding the attachment of the placenta, whether in or over the cervix uteri, and carry it steadily forward, by, over, or through the placenta in search of a foot ; and when he has found it, he will bring it down—and losing no time in useless search for the other foot—he will complete the delivery.

Uterine hemorrhage is a more frequent and fatal occurrence after, than before delivery:—It may arise from retained placenta, or from morbid attachment of a part of it, from want of close contraction of the uterus; or from a too hasty removal of the placenta. If the uterus cannot be provoked to expel its contents by artificial means—by shampooing the abdomen, or irritating the tincae,—the accoucheur will introduce his hand, and not withdraw it until he have secured, (if practicable,) the object for which he introduced it. After this, the uterus may be made to contract by irritating the tincae, or by plugging the vagina, and compressing the abdomen with an appropriate swath.

